# Cancer Resistance to Immunotherapy: Comprehensive Insights with Future Perspectives

**DOI:** 10.3390/pharmaceutics15041143

**Published:** 2023-04-04

**Authors:** Sawsan Sudqi Said, Wisam Nabeel Ibrahim

**Affiliations:** Department of Biomedical Sciences, College of Health Sciences, QU Health, Qatar University, Doha P.O. Box 2713, Qatar

**Keywords:** cancer immunotherapy, resistance, immune checkpoint inhibitors, tumor microenvironment

## Abstract

Cancer immunotherapy is a type of treatment that harnesses the power of the immune systems of patients to target cancer cells with better precision compared to traditional chemotherapy. Several lines of treatment have been approved by the US Food and Drug Administration (FDA) and have led to remarkable success in the treatment of solid tumors, such as melanoma and small-cell lung cancer. These immunotherapies include checkpoint inhibitors, cytokines, and vaccines, while the chimeric antigen receptor (CAR) T-cell treatment has shown better responses in hematological malignancies. Despite these breakthrough achievements, the response to treatment has been variable among patients, and only a small percentage of cancer patients gained from this treatment, depending on the histological type of tumor and other host factors. Cancer cells develop mechanisms to avoid interacting with immune cells in these circumstances, which has an adverse effect on how effectively they react to therapy. These mechanisms arise either due to intrinsic factors within cancer cells or due other cells within the tumor microenvironment (TME). When this scenario is used in a therapeutic setting, the term “resistance to immunotherapy” is applied; “primary resistance” denotes a failure to respond to treatment from the start, and “secondary resistance” denotes a relapse following the initial response to immunotherapy. Here, we provide a thorough summary of the internal and external mechanisms underlying tumor resistance to immunotherapy. Furthermore, a variety of immunotherapies are briefly discussed, along with recent developments that have been employed to prevent relapses following treatment, with a focus on upcoming initiatives to improve the efficacy of immunotherapy for cancer patients.

## 1. Introduction

Cancer is a leading cause of death worldwide, with breast, lung, colon, rectal, and prostate cancers being the most common, according to a 2020 report from the WHO. As of 2020, the cancer incidence and mortality rates were estimated at 19.3 million and 10 million, respectively [1]. For decades, researchers and clinicians have faced the dilemma of treating cancer patients and trying to improve their survival rates. Different approaches have been used in treatment according to the type and stage of the malignancy.

Traditional cancer treatments include chemotherapy and radiation therapy, which together have been the mainstay of cancer treatment for many years [2,3,4]. However, these treatments often have significant side effects and are not always effective in controlling cancer growth. In recent years, there has been a significant interest in developing new cancer therapies that can specifically target cancer cells while sparing normal cells. One of the most promising of these new therapies is immunotherapy.

Immunotherapy is a type of cancer treatment that harnesses the power of the immune system to recognize and destroy cancer cells. The immune system is a complex network of cells, tissues, and organs that work together to protect the body from harmful pathogens and abnormal cells, including cancer cells. Normally, the immune system can recognize and destroy cancer cells, but sometimes cancer cells can evade the immune system and continue to grow and spread [5]. Immunotherapy works by enhancing the ability of the immune system to recognize and attack cancer cells. The discovery of immunotherapy can be traced back to the 1890s, when William Coley first observed the remarkable regression of a sarcoma tumor when triggered by live and inactivated bacteria [6]. In the mid-1990s, the use of monoclonal antibodies was approved by the FDA as a useful anticancer therapy in conjunction with chemotherapy.

There are several different types of immunotherapy, each with its own unique mechanism of action. Some types of immunotherapy work by activating T cells, a type of white blood cell that can recognize and kill cancer cells. Other types of immunotherapy work by blocking immune checkpoints, which are proteins on the surface of T cells that regulate the immune response. By blocking immune checkpoints, immunotherapy can release the brakes of the immune system and allow T cells to attack cancer cells more effectively [7].

Among the most promising types of immunotherapy are checkpoint inhibitors. Checkpoint inhibitors are a type of immunotherapy that block the proteins on T cells that normally prevent them from attacking cancer cells. By blocking these proteins, checkpoint inhibitors can activate T cells and enhance the immune response against cancer cells. Checkpoint inhibitors have been approved for the treatment of several types of cancer, including melanoma, lung cancer, and bladder cancer [8,9,10].

Another type of immunotherapy is CAR T-cell therapy. This type of therapy involves removing T cells from a patient’s blood and genetically modifying them to recognize and attack cancer cells. The modified T cells are then infused back into the patient’s body, where they can seek out and destroy cancer cells. The application of CAR T-cell therapy has shown promise in the treatment of leukemia and lymphoma [11].

Immunotherapy has shown great promise in the treatment of cancer, but it is not without challenges. One of the biggest challenges is identifying which patients will respond to immunotherapy. Not all patients with cancer have an immune system that can recognize and attack cancer cells, and some cancers are more resistant to immunotherapy than others. Due to the genetic instability and heterogeneity of many cancers, cells have developed several mechanisms to escape immune recognition by mitigating the function of T cells in the tumor niche, leading to resistance [12].

Resistance to immunotherapy in cancer is a complex and multifactorial phenomenon that can arise due to various mechanisms. Understanding these mechanisms is critical for developing more effective treatments and improving outcomes for patients with cancer. Hence, the various intrinsic and extrinsic tumor mechanisms causing resistance to immunotherapy are clarified in this review, along with various immunotherapy options.

## 2. The Cancer Immune Cycle

**Figure 1:**
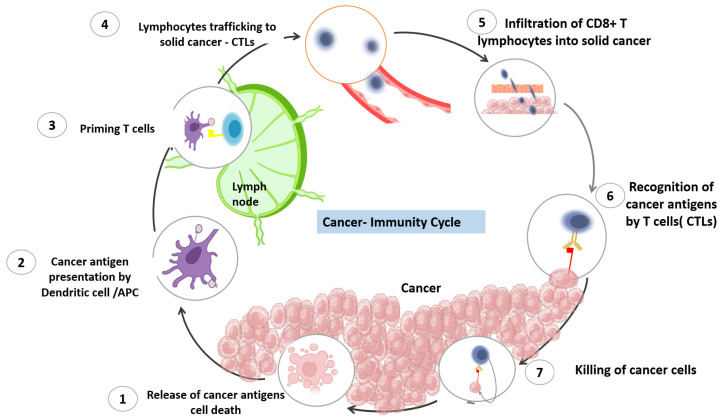
The cancer-immunity cycle. The cancer-immunity cycle is a series of steps through which the immune system passes in order to recognize and attack cancer cells. The cycle includes seven stages, starting with the release of cancer-cell antigens and ending with the destruction of cancer cells by T cells. The first stage involves the release of antigens from dying cancer cells. These antigens are then taken up by dendritic cells, which present them to T cells in the lymph nodes. In the second stage, activated T cells migrate to the tumor site and infiltrate the tumor. The third stage involves recognition of cancer cells by T cells, and the fourth stage involves the activation of T cells to recognize and kill cancer cells. In the fifth stage, T cells work to penetrate the tumor and release cytokines that recruit immune cells to the tumor site. In the sixth stage, immune cells, including macrophages and dendritic cells, infiltrate the tumor and destroy cancer cells. Finally, in the seventh stage, the immune system remembers the cancer cells, creating a lasting immune response that can protect against future cancer development.

This cycle was clearly described by Daniel S. Chen and Ira Mellman in 2013 [7]. The process that starts the cancer immunity cycle is the release of cancer-cell antigens as they are destroyed by chemotherapy. Antigen-presenting dendritic cells then capture these antigens and process them to create peptides that bind to the major histocompatibility complex (MHC) and are then presented to T cells. The CD4+ T-cell receptors can identify the peptide-MHC-II molecules. Subsequently, effector T cells are primed and turned on to react to the tumor antigens that have been presented. The T-cell receptor interacts with its corresponding antigen linked to MHC-I on the surface of cancer cells, causing activated T cells to migrate to the tumor location, infiltrate, and specifically bind to cancer cells. The stimulated T lymphocytes then destroy the cancer cells. Through the activation of a sequence of processes that result in cell death, T cells destroy cancer cells. The cycle is continued, and the anticancer response is amplified by the additional cancer-specific neoantigens that dying cancer cells produce. As the cycle continues, a growing number of tumor antigens are released upon cell death, enhancing the T cells’ immunological response. Furthermore, inhibitory checkpoint proteins, such as CTLA-4 and PD-L1, function at various stages of the cycle to lower the immune activity and, in this way, help to prevent autoimmune reactions. Understanding these stages can help researchers develop new immunotherapies that can enhance the cancer-immunity cycle and improve outcomes for cancer patients. Many immunotherapeutic drugs target specific stages of the cancer-immunity cycle, with the aim of triggering the cancer-immunity cycle without damaging normal cells. The process is demonstrated in Figure 1.

## 3. Innate and Adaptive Immune Responses in the Tumor Microenvironment (TME)

**Figure 2:**
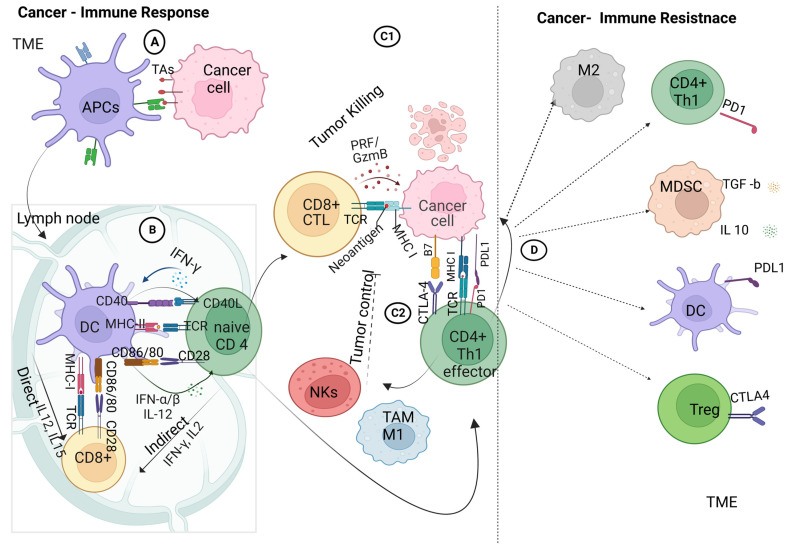
Crosstalk between cancer and immune cells: (**A**) innate adaptive immunity, in which the antigen-presenting cells within the tumor microenvironment take up the tumor-associated antigens and process them for antigen-presenting, priming, and activation immune response; (**B**) adaptive immune response, involving the crosstalk between the dendritic cells and T-cell receptors on naive CD4^+^ T cells and CD8^+^ T cells via the major histocompatibility complex class I/II in the presence of co-stimulatory molecules of CD86/80 on DCs and CD28 on CD4^+^/ CD8^+^ T cells, ending with the activation of effector T cells; (**C1**) interaction of effector cytotoxic T cells with tumor cells within the TME; (**C2**) interaction of effector cytotoxic T cells with tumor cells within the TME and the interaction of effector TH1 cells with tumor cells that trigger the recruitment of tumor-associated macrophages and natural killer cells; (**D**) immune-checkpoint interactions: activation of PD-1 tumor cells to interact with PD-L1 on effector TH1 lymphocytes, ending with the upregulation of immune suppressive cells; macrophages 2 (M2), regulatory T cells (Tregs), and myeloid-derived suppressor cells (MDSCs) produce IL10 and TGF-β1 with further immune-suppressive actions. Created with BioRender.com.

The recognition of cancer cells by the immune system is a promising target in various cancer treatments aimed at eliminating cancer cells from both primary and metastatic sites. This intricate process involves a highly regulated interplay between cellular elements of the innate and adaptive immune systems. Innate immune cells initiate a series of responses, which are followed by adaptive immune responses. The process through which tumor antigens are recognized is illustrated in Figure 2.

When tumor cells die due to treatment or limited blood supply, tumor antigens (TAs) are released. Dendritic cells (DCs), a specific subset of innate immune cells, engulf and process these TAs from the oncogenic mass. These cells act as antigen-presenting cells (APCs), forming a bridge between innate and adaptive immune cells [13].

Next, the DCs move to the lymph nodes, where they present TAs via major histocompatibility complex class II (MHC-II), which binds with receptors on naive CD4 T cells. Co-stimulation occurs when the CD80/86 (B7) on the DCs binds with the CD28 ligand on CD4+ T cells. The autocrine signaling of interleukin-2 (IL-2) is required to trigger and activate the effector-T-cell response, followed by the clonal expansion and differentiation of effector and memory CD4+ T cells [7]. Similarly, naive CD8+ T cells are activated by DCs through the MHC-I complex, with a similar co-stimulation response, leading to the clonal expansion of cytotoxic CD8+ T lymphocytes.

Stimulated CD4+ and CD8+ T lymphocytes may respond to immunogenic signals, such as pro-inflammatory cytokines, which are released from dead tumor or immune cells. Furthermore, DCs express CXCL9 and CXCL10 chemokines to recruit more infiltrating T lymphocytes (ITLs) to the tumor tissue, allowing crosstalk between cancer and immune cells [14].

Infiltrating cytotoxic T cells (CTLs) initiate the immune attack on cancer cells by releasing granzymes, perforin, interferon γ (IFN-γ), and tumor necrosis factor α (TNF-α) [15]. Furthermore, CD4+ cells recognize tumor cells through the interaction of the T-cell receptor with the tumor antigen that is associated with the MHC-I complex on tumor cells in the presence of costimulatory molecules (CTLA4/CD4+ T cells–B7 tumor cells) [16]. Effector CD4+ T cells then provoke tumor-associated macrophages (TAMs/M1) and natural killer cells (NK) in the tumor microenvironment, leading to the mediation of local antitumor immunity with the release of pro-inflammatory molecules [17]. Additionally, the activated NK cells release cytokines targeting tumor cells, leading to subsequent apoptosis.

However, tumor cells modulate the immune response by highly expressing the programmed cell death ligand I (PD-L1) on their surfaces upon interaction with immune cells. The PD-L1 interacts with programmed cell death protein 1 (PD-1) on T cells, leading to the downregulation of T-cell activation and triggering suppressive T-regulatory cells (Tregs) [18]. Furthermore, the membrane-bound transforming growth factor-β1 (TGF-β1) on myeloid-derived suppressor cells (MDSCs) mediates the suppression of NK cells and upregulates more Tregs [19,20]. These modulations are key elements in the initiation of cancer resistance to the immune response.

## 4. Resistance to Cancer Immunotherapy

All cancer therapies aim to overcome the disease and achieve complete recovery. However, despite significant success in treating specific cancer types, many patients still struggle to respond to cancer treatments. Several intrinsic and extrinsic mechanisms contribute to the suppression of the antitumor response following immunotherapy, leading to resistance to immunotherapy. Immune resistance is classified into three types, based on the onset of resistance: primary, adaptive, and acquired resistance.

Primary cancer resistance to immunotherapy refers to a lack of response to immunotherapy in cancer cells at the onset of treatment. This type of resistance can be caused by various mechanisms, such as the activation of immune-checkpoint pathways, which suppress the immune response against cancer cells. Primary resistance can also be attributed to intrinsic factors within cancer cells, such as genetic alterations or changes in protein expression, which make them resistant to the immune response [21]. In contrast to adaptive and acquired resistance, primary resistance is present before immunotherapy is initiated; this is a major challenge in the treatment of many cancer types. Primary resistance to immunotherapy can limit the effectiveness of treatment and is a major focus of ongoing research efforts in the development of new therapies or combinations of therapies that can overcome this type of resistance. Adaptive resistance is characterized by cancer cells adapting within immune-recognition cascades to evade the immune response. This adaptation occurs during the course of treatment and can be considered a form of resistance that develops as a response to treatment. Acquired resistance, on the other hand, refers to the loss of a previously positive response to immunotherapy. This can occur after an initially positive response to treatment, with cancer cells eventually developing resistance to the therapy, leading to relapse. Acquired resistance is often associated with genetic changes that occur within cancer cells over time, leading to a reduced response to the treatment [22]. In contrast to primary and adaptive resistance, acquired or secondary resistance refers to a relapse in treatment and, subsequently, to the primary response. Tremendous work has been undertaken to study and overcome resistance to immunotherapy, with further studies underway to clarify novel mechanisms and new treatment techniques.

The mechanisms associated with resistance to immunotherapy can be categorized into intrinsic factors developed within cancer cells to impede immune recognition and extrinsic factors within the tumor microenvironment, including stromal and immune cells, as well as aberrant blood vessels. In addition to these important etiologies, the effects of other external host factors cannot be ignored when considering resistance to immunotherapy. These factors can include patients’ age, sex, and overall health status, as well as lifestyle factors.

## 5. Tumor Factors (Intrinsic) Associated with Immunotherapy Resistance

**Figure 3:**
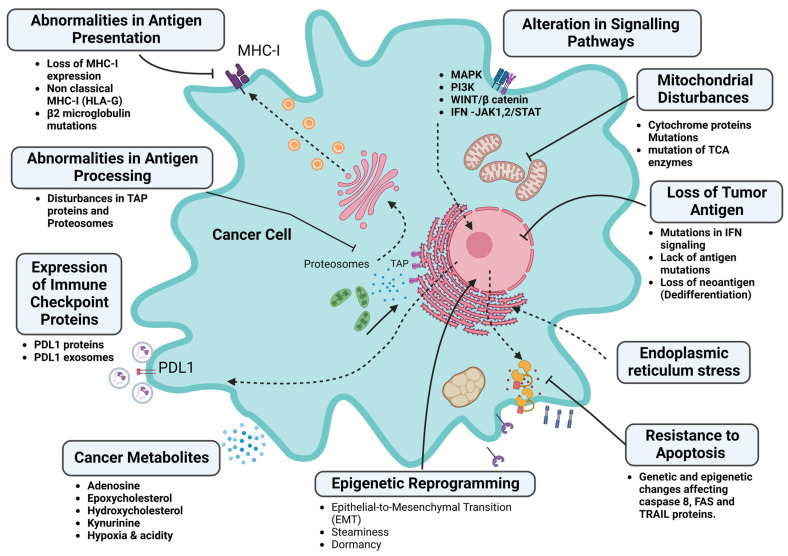
Schematic presentation of the intrinsic tumor factors associated with resistance to immunotherapy. Multiple mechanisms of cancer resistance to immunotherapy, including (I) loss of neoantigen due to the dedifferentiation or lack of antigen mutations, or mutations in IFN signaling; (II) abnormalities in antigen processing, such as endoplasmic reticulum stress, proteosomes, and TAP protein disturbances; (III) aberrant signaling due to the fact of genetic mutations, such as MAPK, PI3, Wnt/β catenin, and interferon JAK1,2/STAT pathways; (IV) abnormalities in antigen presentation due to the loss of MHC-1 expression, nonclassical MHC-I expression, or β2 microglobulin mutations; (V) expression or exocytosis of immune checkpoint proteins, such as PD-L1; (VI) secretion of metabolites by cancer cells, such as adenosine, epoxy cholesterol, hydroxy cholesterol, and kynurenine, in addition to hypoxia and acidosis, leading to the modulation of the tumor microenvironment; (VII) epigenetic reprogramming of cancer cells through stemness transformation or EMT changes; (VIII) resistance to apoptosis by the aberrant expression of intrinsic and extrinsic apoptotic proteins; (IX) mitochondrial disturbances due to mutations in cytochrome proteins or enzymes in the tricyclic-acetic-acid cycle. Created with BioRender.com.

As mentioned above, the success of immunotherapy relies on the recognition of antigenic peptides on the surfaces of tumor cells by antigen-presenting cells, such as dendritic cells, B lymphocytes, monocytes, macrophages, and other immune cells, within the tumor microenvironment [23]. Once these antigenic determinants are processed and presented to CD8+ T lymphocytes, T-helper lymphocytes are activated and infiltrate the tumor environment to initiate an immune response [24]. However, intrinsic factors associated with tumor cells can result in primary or secondary immune resistance to immunotherapy, which can be categorized into factors that lead to the loss of antigenic recognition in tumor cells, factors that interfere with recognition by the immune system, and factors that induce resistance to effector immune response, as demonstrated in Figure 3.

### 5.1. Disorders of the Antigenic Determinants of Tumor Cells

Tumor cells typically express neoantigens, which are tumor-specific antigens that can serve as useful diagnostic biomarkers and potential targets for cancer therapy. A lack of neoantigens is a major pathway of primary resistance to immunotherapy, as it allows tumor cells to evade detection by T cells in the tumor microenvironment. Tumors with high pathogenicity and mutation burden, such as melanoma and renal carcinoma, are more vulnerable to immunotherapy due to their increased antigenicity. High tumor-mutation burden is characterized by multiple somatic mutations in tumor-cell-associated neoantigens, which are present on the surfaces of cancer cells and recognized by the immune system [25]. Microsatellite instability and deficiencies in mismatch repair also contribute to neoantigen presentation. In solid tumors, disturbances in the internal control of DNA replication by the mismatch-repair system can lead to the increased expression of neoantigens due to the presence of various errors in the microsatellites, resulting in enhanced recognition by the immune system [26]. Tumors that cannot express neoantigens exhibit a lack of an effective immune response [27]. The loss of tumor antigens may occur due to the shedding of surface antigens or endocytic-antigen formation, enabling the evasion of immune detection [28]. Tumor cells may also alter antigenic determinants through genetic instability, leading to epitope mutations, causing an antigenic drift similar to that in many viruses [29]. Resistance to immunotherapy may also be attributed to disorders in antigenic processing or presentation mechanisms, including disorders in proteasome formation or alterations in the structure of the major histocompatibility complex due to genetic and epigenetic etiologies, which can lead to the disruption of MHC presentation on the surfaces of cells [30].

The mechanisms associated with the alteration of antigen processing or presentation, such as abnormalities in proteasomes or protein-trafficking channels, such as tapasin, can interfere with protein presentation on tumor cells and antigen-presenting cells [17,26]. Clinical studies have confirmed the association between the loss of these proteins and invasion, metastasis, and diminished cytotoxic T-lymphocyte infiltration in colorectal-cancer patients [30].

The genetic and epigenetic modulation of the genes responsible for the expression of MHC-I proteins may also interfere with antigen presentation, leading to tumor cells’ escape from T-cell-mediated cytotoxicity [30].

Furthermore, the loss of the functional beta 2 microglobulin (B2M) protein may disrupt the MHC class I complex, inducing resistance to immunotherapy. A study on lung cancer showed that 5% of the B2M proteins that were either deleted or mutated resulted in the disruption of antigen-presentation pathways [31]. In addition, multiple-myeloma tumors were associated with the expression of HLA-G, which is a nonclassical MHC-I that interferes with antigen presentation, leading to the evasion of immune recognition, with subsequent resistance to treatment [32].

Disturbances in the signaling pathways involved in antigen presentation can contribute to immune evasion by increasing the expression of PD-L1 or the secretion of chemokines that encourage the recruitment of immunosuppressive lymphocytes [33]. The interferon-gamma cascade plays a role in the effector immune response, which aids in the elimination of tumor cells through the activation of apoptosis. However, the prolonged activation of this cascade can lead to the expression of PD-L1 or PD-1, resulting in immune resistance [34]. Tumor cells may also release the PD-L1 protein into the intercellular space via exosomes, encouraging the transformation of other cancer cells into resistant cells [35]. These adverse events may also arise due to genetic mutations that occur in response to the interferon-gamma cascade, JAK1/2-STAT1. A study confirmed this aberration in the presentation of tumor-surface antigens and resistance to IFN-gamma signaling through RNA sequencing in melanoma cancer cells from patients [36].

In addition to disturbances in the interferon-gamma and antigen-presentation pathways, other signaling pathways have been implicated in resistance to immune therapy. The MAPK, PI3, and WNT signaling pathways, in particular, have been found to interfere with the recruitment and activation of effector immune cells [37]. These pathways may interfere with cascades of antigen processing or presentation that mitigate the recruitment of effector immune cells. The activation of the MAPK signaling pathway was associated with the increased expression of VEGF and IL-8, which are associated with the inhibition of cytotoxic lymphocytes and the recruitment of immunomodulatory cells [38]. Studies have shown that through the inhibition of the MAPK pathway in tumor cells, more infiltrating lymphocytes appear, and a favorable response to immunotherapy is evident with enhanced interferon signaling [39,40]. Consistently, with the activation of intrinsic β catenin/WNT signaling, the dendritic cells are kept out of the tumor tissue, impeding the antigen-presentation process through the inhibition of cytotoxic lymphocytes, leading to the loss of immune response [41].

### 5.2. Factors Modulating the Function of the Immune System

The tumor microenvironment is affected in various ways by the high metabolic demand of cancer cells. One of these effects is the Warburg effect, which results in the buildup of lactic acid and the production of hyaluronic acid and acidosis. Furthermore, the high oxygen demand involved can lead to hypoxia. These factors can contribute to the acquisition of resistance by cancer cells, which can express CD44 to inhibit the function of immune cells in the tumor niche [42]. In one study, the combination of ICI with the mitigation of hypoxia was associated with an enhanced response and better clinical outcomes [43].

Another metabolic effect is the oxidation of cholesterol in tumor cells with the secretion of products, such as hydroxy cholesterol and epoxy cholesterol, that bind to immune cells, leading to the inhibition of dendritic and effector T cells with the activation of modulatory immune cells, leading, in turn, to resistance to immunotherapy [44]. Adenosine, another metabolite released in the tumor environment by tumor cells, attaches to A2A receptors on cytotoxic T lymphocytes, leading to their inhibition with resistance to immunotherapy. Therefore, studies have confirmed the therapeutic benefits of combining ICI and adenosine inhibitors or receptor blockers, with promising clinical outcomes [45].

Cytokines, such as TGF-β, VEGF, and IL10, and other chemokines, such as CCL2, CCL7, CCL8, and CCL13, are also released by tumor cells, leading to interference in immune recognition by the recruitment of immune-modulatory cells, with the inhibition of the proliferation and function of effector cells (discussed further in Section 6).

### 5.3. Factors Associated with Resistance to the Effector Immune Response

These factors are mainly associated with the acquired resistance to immunotherapy, in which, after the recognition of tumor cells by T lymphocytes, tumor cells acquire genetic and epigenetic changes that render them resistant to cell death.

Rough-endoplasmic-reticulum stress and autophagy proteins contribute to immunogenic cancer-cell death. The abundance of LC3B puncta contributes to active autophagy machinery through the infiltration of T lymphocytes associated with sensitivity to immunotherapy [46].

Calreticulin is one of the danger-associated molecular patterns (DAMPs) that are released upon stress into the rough endoplasmic reticulum. This protein is translocated to the cell membrane for immune recognition, acting as a signal for elimination by immune cells, such as dendritic cells and lymphocytes. Disorders in the expression of this protein or its presentation are associated with non-recognition by the immune system [47].

In addition, genetic and epigenetic alterations in the apoptotic machinery may also contribute to resistance to immunotherapy. Several studies have highlighted the link between mitochondrial dysfunction or disorder in the caspase’s proteins or cell-death receptors and acquired resistance to immunotherapy [48,49].

### 5.4. Epigenetic Regulation of Cancer Resistance to Immunotherapy

Genetic instability is one of the hallmarks of cancer cells, and it may potentiate the progression of cancer through different mechanisms, including the evasion of immune recognition. However, secondary resistance to immune therapy is an elusive matter that cannot be merely attributed to the genetic changes in cancer cells; even primary resistance to immunotherapy cannot be fully explained through genetic factors.

Epigenetics is the term used to describe the pathological changes associated with alterations in the expression profiles of many genes without any alterations to the genetic sequence. This may include DNA methylation, histone modification, and other factors contributing to the heterogeneity of cancer cells within a tumor mass. Cancers of germ cells that express a special TAA, known as MAGE antigens, are not expressed with the MHC-I complex. The SSX cancer testis is one of these antigens, and it is downregulated by the hypermethylation of DNA [50]. Upon the use of methylation inhibitors, the expression of these antigens is enhanced, together with immune recognition and response to immunotherapy [51].

Methylation may directly affect the expression of MHC-I and II, thereby interfering with antigen presentation and, thus, impeding immune recognition. Furthermore, methylation inhibitors have shown promising results in breast and colorectal cancers associated with the increased expression of MHC complexes in antigen-presenting cells, with stimulated signaling pathways of immune recognition response and increased sensitivity to immune therapy [52].

An additional immunotherapy evasion mechanism, independent of the initial immune recognition process, is the development of resistance to the cytotoxic granules released by CTLs. This mainly involves the development of resistance to apoptosis in cancer cells due to the methylation of the genes responsible for the expression of apoptotic proteins, such as the caspase 8, FAS, and TRAIL proteins [53]. These changes are reversible through the use of methylation inhibitors associated with increased apoptosis in cancer cells [54]. In addition, epigenetics may also modulate the function of immunosuppressive cells in the tumor microenvironment, including regulatory T cells and myeloid-derived suppressor cells, which cause resistance to immunotherapy through the secretion of inhibitory cytokines, including IL-35 and TGF-β [55,56].

The epithelial–mesenchymal transition (EMT) is another demonstration of epigenetics that occurs during embryonic development and wound healing, in which epithelial cells lose their polarity and cell-to-cell contacts and gain mesenchymal properties, such as motility and invasiveness. The EMT also plays a crucial role in cancer progression, in that it promotes tumor invasion, metastasis, and therapy resistance. Recent studies have implicated EMT in resistance to immunotherapy in various types of cancer. One of the mechanisms through which EMT promotes immunotherapy resistance is through the downregulation of immune checkpoints on cancer cells. The EMT can downregulate PD-L1 expression on cancer cells, making them less susceptible to PD-1-blockade therapy [57]. Another way in which the EMT confers immunotherapy resistance is by altering the tumor microenvironment. The EMT can increase the recruitment of immune-suppressive cells, such as regulatory T cells (Tregs) and myeloid-derived suppressor cells (MDSCs), into the tumor microenvironment. Tregs can inhibit the activity of effector T cells, while MDSCs can inhibit the activity of both T cells and natural killer (NK) cells. The EMT can also upregulate the expression of cytokines, such as interleukin-6 (IL-6) and interleukin-8 (IL-8), which can increase immune suppression and angiogenesis [58]. In addition, the EMT can also increase the expression of drug-efflux pumps, such as ATP-binding cassette transporters (ABC transporters), which can pump out chemotherapeutic drugs and immunotherapeutic agents, reducing their effectiveness [59]. In one study, Anushka et al. were able to demonstrate that breast-cancer cells with an epithelial phenotype had a higher expression of the MHC-I complexes and lower expression of PDL-1, and the tumor stroma contained cytotoxic T cells and M1 macrophages [60]. In contrast, the mesenchymal versions of the cancer cells had lower MHC-I complexes and higher PDL-1 expression, and their environment was populated with exhausted cytotoxic T cells and other immunosuppressive cells. Nonetheless, the epithelial cancer cells were highly sensitive to the anti-PD-L1 or anti-CTLA-4 immunotherapy, whereas the mesenchymal breast-cancer cells were more resistant to these treatments. Overall, these findings suggest that the EMT plays a significant role in immunotherapy resistance in cancer. Thus, targeting the EMT pathways in combination with immunotherapy may provide a novel approach to improve the response to immunotherapy in cancer patients.

Given all this evidence, epigenetic alterations are largely involved in the resistance to immunotherapy. Based on this, other treatment options have emerged for cancer patients, such as anti-TGF-β or methylation inhibitors, which could provide more cures when combined with other immunotherapies, such as ICIs.

## 6. Extrinsic Factors Contributing to Tumor Resistance against Immunotherapy 

**Figure 4:**
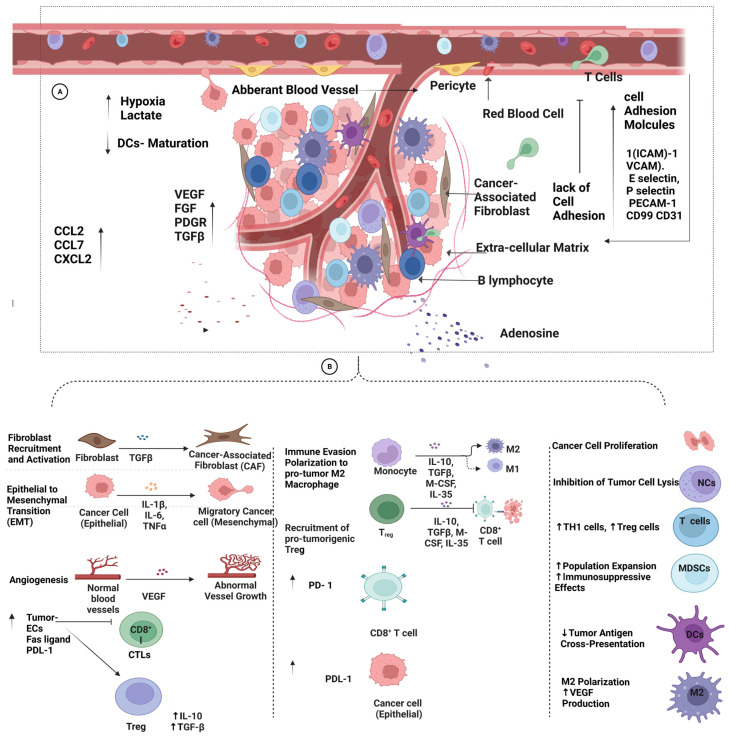
Schematic presentation of the extrinsic factors contributing to tumor resistance against immunotherapy. (**A**) Tumor growth and the hypoxic effect on the TME trigger the expression of immunosuppressive and proangiogenic factors, such as TGF-B, VEGF, EGF, FGF, and PDGF. Various vascular changes are mediated by VEGF and immunosuppressive factors, including dysfunctional blood vessels and a lack of both epidermal cells and cell-adhesion molecules (ICAM, P-selectin, and E-selectin). These changes prevent the infiltration of immune cells into the TME and downregulate the immune response. Furthermore, aberrant blood vessels lead to a more stressful and enriched environment, with acidic and hypoxic factors that increase VEGF and prevent DC maturation. Chemokines, such as CCL2, CCL7, and CXCL2 abate/elevate tumor–immune responses and recruit MDSCs, which support tumor growth via TGF-ß. (**B**) In the tumor microenvironment (TME), further immunosuppressive changes have been observed, such as elevated PD-L1 levels in tumor cells and myeloid-derived suppressor cells (MDSCs), the activation of oxidative and fatty acid metabolism pathways in inactive and resting T cells, and a shortage of glucose that hinders the function of IL-2-stimulated T cells and interferon-gamma (IFN-γ) production.. The upregulation of the TME’s metabolites, such as adenosine, which is produced by TAMs and Tregs, acts as a mediator of immunosuppression. Created with BioRender.com.

The extrinsic mechanisms of resistance to immunotherapy largely depend on the different constituents within the TME that interfere with the presence and function of tumor-infiltrating lymphocytes (TILs), including CD4+, CD8+, B lymphocytes, and natural killer cells [61]. This interference, manifested within the TME, is exploited by tumor cells to suppress the immune response, favoring the presence of cancer-promoting constituents. The description of various extrinsic factors in the immune resistance of tumors is illustrated in Figure 4.

At the tissue level, several immunosuppressive cells in the tumor microenvironment interfere with T-cell activation through different mechanisms [12,62]. These include regulatory T cells, N2 neutrophils, myeloid suppressor cells, M2 macrophages, and tumor-associated fibroblasts [63,64,65,66]. These cells may induce immunotolerance through the expression of immune-checkpoint proteins, such as PD-1 and CTLA-4, on the surfaces of cells, interfering with the immune response at the tumor site, in addition to the secretion of immunomodulators, such as interleukin 10, interleukin 35, and TGF-β [67]. These immune mediators were found to establish an immunosuppressive environment with the apoptosis of dendritic cells, the maturation of monocytes into M2 macrophages, and the polarization of neutrophils to the N2 type, limiting the antineoplastic phenotype of N1 neutrophils [68]. Additionally, N2 neutrophils, myeloid suppressor cells, and M2 macrophages may also interfere with the immune response through the secretion of reactive oxygen species, arginase-1, and inducible nitric-oxide-synthase enzymes that enhance the proliferation and function of regulatory T cells while inhibiting the priming of effector T cells [69].

In addition, TGF-β may also interfere with T-cell priming through the suppression of T-cell proliferation and dendritic-cell function [70]. In addition, TGF-β may reprogram the stromal fibroblasts in the tumor microenvironment into an immune modulatory phenotype of cancer-associated fibroblast that further represses the immune response through the secretion of TGF-β and IL6, leading to the inhibition of antigen-presenting dendritic cells and T-cell priming, eventually leading to suppressive immune stroma [71].

Cancer-associated fibroblasts may also reorganize the extracellular matrix, causing dense stroma that limit the penetration of effector cells, in addition to the modulation of cytokine and chemokine gradients, limiting the attraction of effector T cells [72]. Cancer-associated fibroblasts may also express immune-checkpoint ligands on their cells’ surfaces, leading to the inhibition of T-cell function [73].

### 6.1. Tumor-Infiltrating Lymphocytes

Tumor-infiltrating lymphocytes (TILs) play a pivotal role in the immune response within the TME, in which the infiltrated lymphocytes adopt different phenotypes that either support the growth of tumor cells (T regs) or contribute to the elimination of cancer cells (cytotoxic T cells). Tumor cells may control the function of lymphocytes within the TME through several cytokines, such as TGF-ß and IL-2, leading to immunosuppression and tolerance, by converting the naive T cells into Treg cells, or the countering of autoimmunity, by inducing Th17 cells [74].

A study on advanced colorectal cancer (CRC) demonstrated the depletion of T-cell infiltration through increased TGF-ß levels, which provoked immune suppression. Accordingly, with anti-TGF-ß therapy, the immunosuppressive changes were reversed, provoking the infiltration of more cytotoxic immune cells. In addition, the anti-TGF-ß therapy potentiated the anti-PD-1/PD-L1 therapy synergistically and improved the immune response of CRC [75].

### 6.2. Chemokines

Chemokines are primarily known for chemoattracting immune cells, regulating tumor proliferation, and mediating angiogenesis and metastasis [76]. Chemokines released into the TME may further modify the immune response within the TME.

Chemokines, such as CXL9 and CXL10, orchestrate the movement of Th1 cells into the TME via the chemokine receptor CXCR3+, in which case Th1 acts strongly against tumors, arrests cellular proliferation via IFN gamma, and reduces the angiogenesis in the TME [77]. In pancreatic-adenocarcinoma and colon-cancer patients, the infiltration of Th1 cells was associated with a better prognosis and was considered a survival marker [78].

The CCL-4 is associated with favorable responses in the TME due to its recruitment of antigen-presenting dendritic cells [79]. Furthermore, DCs, macrophages, and neutrophils contribute to the modulation of the immune response in the TME, and they are recruited by different chemokines to exhibit either pro- or antitumor functions.

On the other hand, other chemokines are associated with the infiltration of tumor sites by myeloid-derived suppressor cells (MDSCs), including CCL2 and CXCL8 (IL-8) [79]. Other chemokines and chemokine receptors that increase the recruitment of MDSCs include CCL7, CCL8/CCR2, CCL5/CCR5, CXCL1, CXCL2, CXCL5/CXCR2, and CXCL12/CXCR4 [80]. The presence of MDSCs may support the establishment of a suppressed immune environment at the tumor site, with fewer functional CD8^+^ T lymphocytes, and may support tumor growth in different ways, such as by producing angiogenesis factors, including transforming growth factor beta, platelet-derived growth factors (PDGRs), fibroblast growth factors (FGFs), and epidermal growth factors (EGFs) [81]. Moreover, these chemokines mediate the migration of endothelial cells to the TME, contributing to the immunosuppressive effects. This observation was demonstrated in breast-cancer cells in which CCL5 and VEGF were expressed in lymphatic endothelial cells in response to factors released by triple-negative breast-cancer cells associated with angiogenesis and metastasis [82].

### 6.3. Vascular-Tumor Microenvironment

Among the immunosuppressive factors released by tumor tissues is the vascular endothelial growth factor (VEGF), which is associated with changes to the structure and function of blood vessels, as shown in Figure 4. In this process, dysfunctional tumor blood vessels are developed with defective endothelial cells (ECs) that express low levels of cell-adhesion molecules [83]. Typically, the infiltration of lymphocytes into the TME begins with lymphocytes’ adhesion to ECs that roll and migrate through endothelial blood vessels. The process of transmigration largely depends on adhesion molecules, which are expressed either on lymphocytes through pro-inflammatory cytokines, such as TNF α, IL-1B, and LPS, or on endothelial cell surfaces, including intercellular adhesion molecule-1 (ICAM)-1, vascular cell adhesion molecule-1 (VCAM), E-selectin, P-selectin, platelet–endothelial cell adhesion molecule-1 (PECAM-1), CD99, and CD31 [84].

The lack of adhesion molecules prevents immune-cell infiltration into the TME and disturbs endothelium–T-cell interaction, hindering T-cell adhesion and diapedesis through blood vessels. A study on advanced ovarian cancer investigated the relationship of VEGF and receptor expression with the infiltration of MDSCs through a microarray and the functional inhibition of VEGF. The study confirmed the involvement of the infiltration of MDSCs in VEGF signaling, which was reversed with anti-VEGF therapy [85].

Furthermore, vascular impermeability might resist immunotherapy access or its delivery into the TME and develop tumor resistance. In addition, tumor ECs may express PD-L1 and Fas ligands, which disable the CTLs and trigger the formation of more regulatory T cells within the TME to maintain immunosuppressive status [86]. Moreover, tumor ECs produce immunosuppressive factors (agniocrine) that inhibit T-cell survival/activation and provoke immunosuppressive phenotypes of tumor-associated macrophages (TAMs) via IL-6, TGF-ß, and (VEGF) [85].

Furthermore, aberrant tumor vessels create a stressful environment enriched with acidic and hypoxic factors. This is due to the high demand for glucose by tumor cells, resulting in acidosis due to the high levels of lactate, as well as oxygen depletion, within the TME, which can enhance the establishment of an immunosuppressive TME, as discussed above. Notably, the hypoxic state is dependent on hypoxia-inducible factor 1 alpha, (HIF-1a), which increases the expression of VEGF via the MAPK/ERK pathway [87]. Moreover, tumor ECs use CCL2, macrophage costimulatory factor (M-CSF), and VEGF to support their growth and the development of metastasis. VEGF binds to their receptors and prohibits the maturation of DCs and antigen presentation [88]. The vascular microenvironment may induce the expression of PD-L1 on tumor cells, CTLA-4, T-cell immunoglobulin 3 (TIM-3), and PD-1 on immunosuppressive cells, blocking the immune response.

Interestingly, PD-L1-negative tumors respond well to PD-L1 inhibitors, which might be attributed to the contribution of PD-L1 therapy to the regulation of host immune cells as an anti-angiogenic therapy [89]. Antiangiogenic therapy reprograms aberrant normal vessels by inducing the maturation of DCs and antigen presentation, as well as upregulating the expression of cell-adhesion molecules, which facilitates immune-cell infiltration into the TME. Moreover, a study demonstrated that the use of antiangiogenic therapy in breast cancer upregulated the expression of PD-L1 on the surfaces of ECs and tumor cells and increased the sensitivity with anti-PD-1 therapy [88].

### 6.4. Immuno-Metabolic Products

In the TME, immune-cell metabolism may further contribute to the pathogenesis of resistance. Due to the high energy demands of tumor cells, immune cells within the TME may alter their metabolism by expressing more glucose transporters with high oxygen demand [90]. A hypoxic TME triggers the increased expression of HIF1α in tumor cells and MDSCs, which results in the upregulation of the PD-L1 and glycolytic pathways in both cells, while the high expression of HIF1α in TILs provokes PD-1 expression. Hence, the interaction between PD-1T cells and PD-L1 tumor cells activates T-cell exhaustion and immune resistance.

In addition, high lactate levels in the TME may suppress TILs by releasing reactive oxygen species (ROS), adding more stress to T cells [91]. On the other hand, immune cells (naive and resting T cells) may exhibit adaptations within oxidative and fatty-acid metabolisms to increase glucose uptake. Consequently, CD4^+^ and CD8^+^ effector cells upregulate the expression of glycolysis and tricarboxylic-acid-cycle-related genes. Furthermore, insufficient glucose levels lead to the inactivation of nuclear factor of activated T cells (NFATs). This inactivation negatively impacts the persistence of IL-2-stimulated T cells and suppresses the production of IFN-gamma.

As mentioned above, metabolic reprogramming induces higher levels of lactate, which results in the expression of CD44 and hyaluronic-associated metastasis [21]. Additionally, in tumor cells, cholesterol is esterified to epoxy cholesterol and hydroxycholesterol, which are associated with the maturation of Th17 inhibitory immune cells, and the deactivation of DCs results in a lack of antigen presentation to T cells [21]. Interestingly, these changes were reversed with a cholesterol-esterification inhibitor, which modulated the immune response and enhanced CD8^+^ cytolysis.

In addition, MDSCs and tumor cells produce indoleamine 2,3-dioxygenase (IDO) enzymes, which utilize tryptophan and produce an immunosuppressive metabolite known as kynurenine [85]. Accordingly, in recent clinical trials, sarcoma and breast cancers targeting IDO metabolism by inhibiting tryptophan production showed a fair level of success by activating CD8+ and suppressing the Tregs within the TME [90].

Notably, within the TME, adenosine metabolites are produced from ATP via CD39 and CD73 on TAMs and Tregs. Adenosine is known as a mediator of immunosuppressive and anti-inflammatory response and is reducible to functional neutrophils and macrophages. Adenosine binds to the A1, A2a, A2b, and A3 receptors on cells, such as effector T cells. Adenosine mainly interacts with A2a receptors on T-effector cells and upregulates the checkpoint expression of PD-1. Tumor cells upregulate CD38 to produce adenosine, which encourages metastasis by binding to tumor A2B receptors, causing defects in the T-cell-signaling pathway via the interruption of the IL2-induced phosphorylation of STAT 5a and STAT5b in primary and CTLL-2 T cells. Thus, utilizing adenosine receptor antagonists might improve the responsiveness of IL-1, T-cell signaling, and the production of proliferative functional T lymphocytes [92,93].

### 6.5. Tumor-Associated Macrophages (TAMs)

Tumor cells polarize TAMs to anti-inflammatory cells (M2), which produce IL-10 and TGF ß and express PD-L1, which in turn interact with NKs and T cells via the PD-1 and PD-L1 checkpoints [83]. Studies have demonstrated the effects of these epigenetic molecules on the TME, which lead to the reversal of the tumor immune response. In gastric cancer, one study showed the contribution of the epigenetic factors of the DNA methyltransferase (DNMT) family (DNMT1, PRMT, and KDM6B), which led to the upregulation of M2 and TAMs within TAMs. With the epigenetic modification of the HMG-box transcription factor, TOX can restore effector T cells during exhaustion. However, in chronic inflammation, TOX triggers T-cell exhaustion [94].

## 7. Host Factors Associated with Immunotherapy Resistance

**Figure 5:**
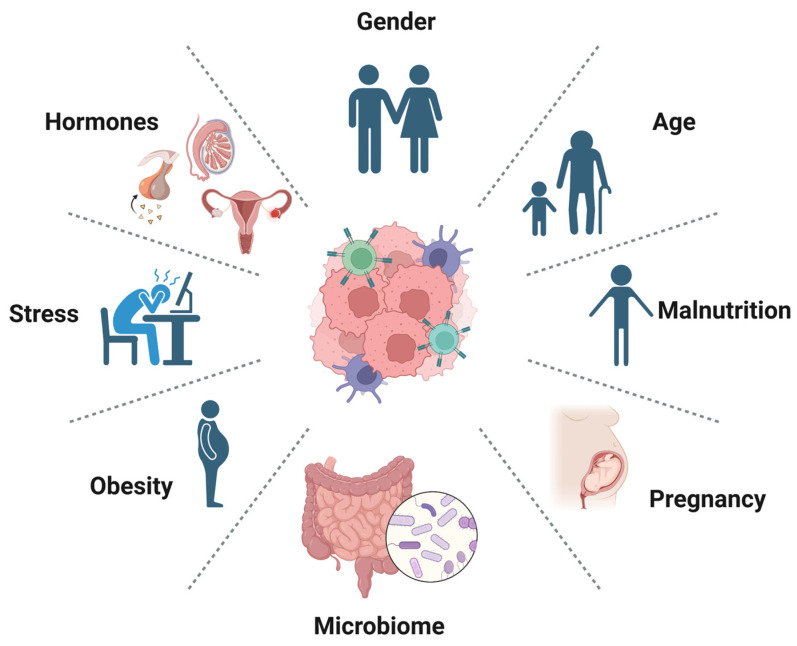
Host factors associated with resistance to immunotherapy in cancer patients. (I) Gender is a factor due to the physiological differences between the innate and adaptive immune systems. (II) Age is indicated in studies as the elderly are more likely to develop resistance than their younger counterparts. (III) Malnutrition may interfere with cell-mediated immune responses, which may allow individuals to develop resistance to immunotherapy. (IV) Pregnancy-associated physiological adjustments, including immunotolerance, could interfere with immune recognition and, subsequently, lead to resistance. (V) Microbiomes, such as *Bifidobacterium*, *A. muciniphila*, *Faecalibacterium*, and *Bacteroidales*, were associated with the enhancement of the immune response in cancer patients, while imbalances in the microbiota were abundant in cancer patients developing resistance to immunotherapy. (VI) Obesity was shown to increase resistance to immunotherapy using animal models of cancer. (VII) Stress is an environmental factor associated with the modulation of immune responses that weaken adaptive immune responses, leading to the failure of the response to immunotherapy. (VIII) Hormones, such as testosterone, estrogen, and progesterone, also modulate the innate and adaptive immune responses, leading to variable responses to immunotherapy in both genders. Created with BioRender.com.

Multiple host factors are associated with resistance to immunotherapy at the systemic and tissue levels, as shown in Figure 5. At the systemic level, these include old age, gender, BMI, microbiome, and hormonal factors [95,96,97].

Based on the findings of many studies and through a meta-analysis, it appears that patients who are older than 65 years tend to have a greater response to immunotherapy than younger individuals [98]. However, the exact mechanism behind this is not well understood. Gender is another important factor that could affect the response to immunotherapy, based on biological, hormonal, and immunological differences [99]. However, this observation requires careful investigation due to the presence of confounding factors, such as tumor stage, race, histological type and biomarkers of cancer, smoking status, and menopause, which need to be considered.

Obesity is another host factor that is associated with resistance to immunotherapy. This was validated in many animal studies in which animal models of obesity were associated with immunosuppressive responses associated with poor infiltration into the tumor tissue of cytotoxic lymphocytes [100].

The microbiome, on the other hand, is an influential factor associated with tumor progression and response to immune therapy. Germ-free animals were found to have a higher likelihood of acquiring cancers, and their tumors were more aggressive compared to those of controls [101]. Through many in vivo studies, the role of the microbiota was found to enhance the response to immune therapy, especially CTLA-4 blockage and PD-L1, with microorganisms such as *Bacteroidales*, *Burkholderiales*, *B. fragilis*, *B. thetaiotaomicron*, *B. cepacian*, *Bifidobacterium* species, *Bifidobacterium*, *Akkermansia*, *Enterococcus*, *Bifidobacterium*, *Faecalibacterium*, *Lactobacillus johonsonii*, and *Enterococcus* [101].

The mechanism behind the immune response involving these bacteria is attributed to the release of short-chain fatty acids, such as butyrate, which directly modulate the expression of transcription factors in tumor cells and immune cells, such as lymphocytes, macrophages, and dendritic cells [102]. In addition, other substances released by the microbiota, such as pyridoxine, vitamin B, and ferrichrome, could affect cancer cells directly, causing apoptosis or modulating the tumor environments through cytokines, such as IL-6 and IFN-γ, leading to the maturation of regulatory T lymphocytes.

Pregnancy is a unique physiological state in which the maternal immune system undergoes significant changes to accommodate the developing fetus. These changes, including alterations in the balance of immune-cell subsets and cytokine production, have the potential to affect the immune response to cancer. Recent studies suggest that pregnancy may affect the efficacy of immunotherapy and contribute to the development of cancer.

During pregnancy, the immune system undergoes significant changes, including an increase in regulatory T cells (Tregs), which may contribute to immunosuppression and the development of cancer [103]. Tregs play an important role in maintaining immune tolerance to self-antigens and preventing autoimmunity. However, they can also inhibit the antitumor immune response, thereby encouraging tumor growth and metastasis. Studies have shown that the increased levels of Tregs during pregnancy may contribute to the development of cancer or interfere with the efficacy of immunotherapy. For example, a recent study showed that breast-cancer patients who had a history of pregnancy had higher levels of Tregs in their tumors, which were associated with a worse prognosis [104]. Further studies are needed to determine the mechanisms underlying these effects and to identify potential strategies to improve outcomes in pregnant patients with cancer. Understanding the complex interplay between pregnancy and cancer immunity may also provide insight into the broader field of immunotherapy.

Stress has been shown to activate the hypothalamic–pituitary–adrenal (HPA) axis and the sympathetic nervous system (SNS), leading to the release of stress hormones, such as cortisol and epinephrine [105]. These hormones can have immunosuppressive effects, including the inhibition of T-cell activation and proliferation, an increase in T-cell apoptosis, and the suppression of natural killer (NK) cell function [105].

In a recent study, researchers examined the relationship between psychological stress and the response to immunotherapy in patients with advanced non-small-cell lung cancer (NSCLC) receiving immune-checkpoint inhibitors (ICIs) [21]. They found that the patients who reported higher levels of psychological stress had a lower objective response rate (ORR) and progression-free survival (PFS) than the patients with lower levels of stress. Furthermore, the study found that stress was associated with a decrease in peripheral blood CD8+ T cells and an increase in regulatory T cells (Tregs), which can inhibit the antitumor immune response [21].

Another study investigated the association between stress and the response to pembrolizumab, an anti-PD-1 antibody, in patients with melanoma [106]. The study found that the patients who reported higher levels of perceived stress had a poorer response to pembrolizumab, as measured by reduced tumor shrinkage and progression-free survival. The study also found that stress was associated with a decrease in peripheral-blood CD8+ T cells and an increase in myeloid-derived suppressor cells (MDSCs), which can inhibit the antitumor immune response [106].

While these studies suggest a potential link between stress and resistance to immunotherapy, it is important to note that they are relatively small, and further research is needed to confirm their findings in larger patient populations. It is also important to consider that stress is a complex and multifaceted phenomenon that can be difficult to measure accurately. Nonetheless, these studies highlight the potential impact of psychosocial factors on cancer immunotherapy and the importance of considering the whole patient in the treatment of cancer.

Malnutrition can weaken the immune system and impair the ability of immune cells to recognize and eliminate cancer cells. As a result, cancer patients who are malnourished may have a reduced response to immunotherapy. Studies have found that malnourished cancer patients have lower levels of immune cells, such as T cells and natural killer cells, which are critical for fighting cancer [107]. Malnourished patients may also have lower levels of cytokines, which are signaling molecules that help activate the immune response [108]. Therefore, addressing malnutrition in cancer patients is essential to optimize their responses to immunotherapy. Nutritional interventions, such as dietary changes and supplements, can help improve the immune system and enhance the efficacy of immunotherapy.

## 8. Models of Cancer Immunotherapy

**Figure 6:**
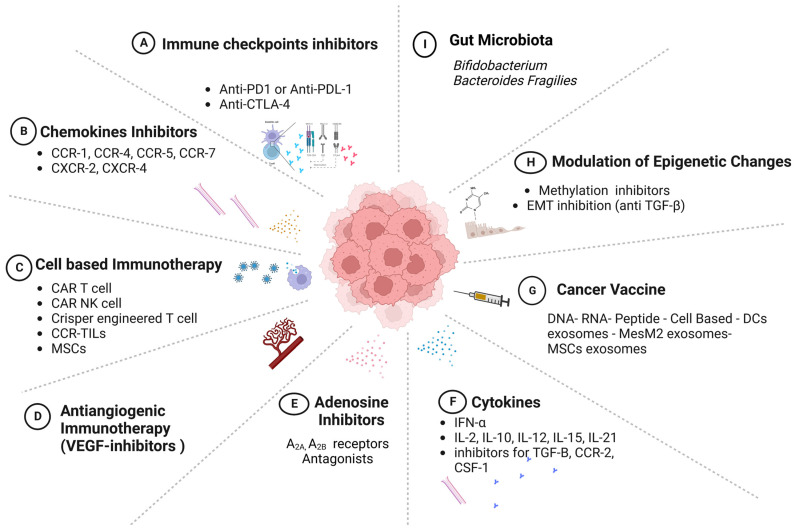
Cancer immunotherapies. Different approaches are highlighted. (**A**) Immune-checkpoint inhibitors: anti-PD-1/anti-PD-L1 and anti-CTLA4. (**B**) Blockade of chemokines/chemokine receptors within the TME to inhibit cancer growth or the recruitment of immune-suppressor cells. (**C**) Cell-based immunotherapies, such as the use of genetically modified T-cell receptors to recognize tumor-antigen-bounded MHC molecules and block cancer-escape pathways, in addition to NKs, MSCs, and TIL cells that are genetically modified to trigger the proliferation and development of immune cells. (**D**) Antiangiogenic inhibitors reprogram aberrant blood vessels and facilitate the infiltration of the TME by immune cells. (**E**) Metabolite inhibitors, such as adenosine inhibitors (A2a- and A2b-receptor antagonists). (**F**) Cytokine therapy provoking immunity in melanoma and renal cancer (IL-2). (**G**) Different forms of cancer vaccine to encounter immune cells. (**H**) Modulation of epigenetic changes, such as the epithelial–mesenchymal transition and gene methylation. (**I**) Gut microbiota to improve the response to ICI therapy. Created with BioRender.com.

Immunotherapy has led to the development of multiple lines of treatment that target different components of the immune system, with the goal of enhancing the recognition and elimination of cancer cells. The various modalities of immunotherapy are illustrated in Figure 6. They include monoclonal antibodies, cytokines, checkpoint inhibitors, adoptive cellular immunotherapy, chemokine inhibitors, antiangiogenic immunotherapy through VEGF inhibitors, adenosine inhibitors, modulators of epigenetic changes, vaccines, and gut-microbiota therapies [109,110].

### 8.1. Immune-Checkpoint Inhibitors

The most widely used form of immunotherapy is that of immune-checkpoint inhibitors (ICIs), which presented a breakthrough in the management of aggressive malignancies based on the blocking the “off signal” over T-cell receptors [111,112]. Predominantly, immune-checkpoint proteins are expressed on the surfaces of T cells, including cytotoxic T-lymphocyte-associated protein 4 (CTLA-4), programmed death-1 (PD-1), and programmed death ligand-1 (PD-L1), which control the duration of the immune response and restrict self-tolerance, as shown in Figure 1 [12,113]. Several agents were developed to target the signaling axes of different immune-checkpoint proteins, including CTLA-4 and PD-1 in T lymphocytes and the PD-L1 ligand in cancer cells. In 2011, anti-CTLA-4–B7 monoclonal antibodies (ipilimumab) were approved by the FDA as anticancer drugs for advanced melanoma and lung [109] and renal carcinoma [112,114]. The results of studies on this topic were promising and provoked the development of further inhibitors, including anti-PD-1 (pembrolizumab and nivolumab) and anti-PD-L1 (atezolizumab and durvalumab), which are used to treat breast, gastric, and hepatocellular cancers. In animal models and clinical trials, the combination of a CTLA-4 and PD-1/PD-L1 blockade demonstrated a synergistic effect in eliminating cancer cells, leading to improved survival outcomes for cancer patients [115,116].

In several trials, adverse effects of immune-checkpoint inhibitors were reported due to the amplified inhibition associated with autoimmune responses, leading to life-threatening conditions due to the inhibition of tolerance responses and the resultant damage within internal organs, such as the liver, kidneys, lungs, and intestines [117]. In addition, the responses varied among cancer patients according to the type, location, stage of the cancer, and the expression patterns of the ICP proteins. Therefore, indicators such as microsatellite instability (MSI), PD-L1, and tumor mutational burden (TMB) were approved by the FDA as predictive markers in cancer patients and used before treatment to predict patients’ responses to specific treatments [118].

Other immune-checkpoint inhibitors launched with FDA approval to improve the prognosis of patients not responding to traditional ICIs include treatments targeting the lymphocyte-activation gene-3 (LAG-3), which is involved in the interaction between lymphocytes and cancer cells, dendritic cells, and myeloid-derived suppressor cells, leading to the suppression of T-cell function and the inhibition of cytokine secretion [119]. Further developments included therapies targeting T-cell immunoglobulin and mucin domain 3 (TIM-3) on T cells that interact with gal9 in cancer cells, leading to the exhaustion of T cells and natural killer cells [120]. Inhibitors of the human endogenous retrovirus-H long-terminal repeat-associating protein 2 (HHLA2) are promising treatments that could help patients with primary resistance to anti-PD1/PDL-1, such as in melanoma and hepatic cholangiocarcinoma [121]. In addition, V-domain immunoglobulin-containing suppressor of T-cell activation (VISTA)-based therapies help to alleviate the suppressive effect on T cells upon interaction with suppressor myeloid cells, in addition to their promising therapeutic effects on autoimmune diseases [122].

### 8.2. Chemokine Therapies

Chemokines are categorized based on their effects within the TME on pro-tumor or antitumor molecules. Interestingly, various chemokines are produced from cancer cells and interact with the receptors on these cells, enhancing their proliferation or binding with endothelial cell receptors within the TME to attract immune-suppressor cells, such as myeloid-derived suppressor cells (MDSCs) and M2 tumor-associated macrophages (TAMs). Thus, chemokines are promising targets in cancer immunotherapy that can inhibit or augment specific immune responses. For instance, either chemokines or their receptors might be inhibited to augment the antitumor response. Several treatments have been developed, including chemokine antagonists, to inhibit pro-tumorigenic effects and increase the number of antitumorigenic chemokines.

Chemokine treatments were also found to synergistically augment the response to adoptive cell therapy, with more chemokine receptors binding to chemokine ligands, to target the infiltration into the tumor niche [123]. The treatments used for chemokines/chemokine receptors include neutralizing antibodies, chemokine inhibitors, cancer vaccines, and specific viral vectors [124]. For instance, the use of neutralizing antibodies and inhibitors against pro-tumorigenic chemokines, such as cartlumab antibodies, was applied to target CCL2-mediated TAM infiltration in breast and prostate tumors [123]; however, this line of treatment had a limited response. The anti-CCR4 antibody was approved as a treatment for relapsed and refractory adult T-cell leukemia–lymphoma [81], whereas BPRCX807 was used in hepatocellular carcinoma as a CXCR4 antagonist and showed promising effects, including reduced metastasis, tumor growth, and angiogenesis [125].

Furthermore, CXR4, CXR1/2, CCR5, and CXCR3 and their ligands have been applied as cancer immunotherapies. Further clinical trials utilized specific neutralizing antibodies or inhibitors against chemokines. In 2021, a phase III clinical trial on acute myeloid leukemia (AML) patients showed promising responses when the CXCL12 chemokines were inhibited by DSTAT (dociparstat sodium) in combination with chemotherapy [123]. A recent study on the role of chemokines in multiple-myeloma tumors confirmed the high expression of CCL3 (osteoclast-activating factor) by cancer cells. Consequently, neutralizing the CCL3 receptors in vivo deterred metastasis and limited tumor progression [126].

Consistently, the use of combined therapy has gained in popularity, especially for advanced stages of malignancies with resistance to mainline treatments. The use of such treatment regimens is supported by animal studies, which showed an enhanced response to ICIs in xenograft models of melanoma, breast, and prostatic cancers [127]. The use of chemokines, including CXCL1 and CXCL9, in adjuvant therapies is a promising line of treatment. Currently, a phase II clinical trial is underway to evaluate the use of a combination of ICIs and antibodies that neutralize chemokines. However, there are difficulties in regulating the effects of chemokine treatments on both tumor and immune cells in the TME. These treatments have the potential to affect various types of immunological cell, with unpredictable and challenging consequences within the TME, making it difficult to predict or control the outcomes.

### 8.3. Adenosine-Targeting Therapies

Another potential metabolite within the TME that acts as an immunosuppressive factor is adenosine, which results from the conversion of ATP to AMP by the cell-surface enzyme, C39, in Tregs and B cells, or CD73, in stromal and mesenchymal B cells, fibroblasts, and endothelial cells [128]. In the tumor microenvironment, the secretion of adenosine can increase by up to ten times its normal levels, resulting in immunosuppressive effects that include the inhibition of T-cell proliferation and cytokine release, as well as hindering dendritic-cell maturation and the recruitment of immune-suppressive cells. This is achieved through the binding of adenosine to the A2AAR receptor [128]. To counter these effects, adenosine receptors can be blocked; this has the potential to downregulate the immunosuppressive effects and trigger an immune response. Although various adenosine antagonists have been tested in clinical trials, they have only shown mild responses. However, their efficacy can be improved when combined with other cancer therapies [129].

### 8.4. Cell-Based Immunotherapy—Adoptive Cellular Transfer (ACT)

This treatment emerged as a promising approach in immunotherapy in the 1950s. The first form of ACT was the use tumor-infiltrating lymphocytes (TILs) isolated from cancer-patient specimens, enriched, and then reinfused back into the patients [130]. Despite the demonstration in previous studies of the variable effectiveness of using TILs in ovarian cancer, renal-cell carcinoma, and metastatic cases, TILs have not achieved more than a 50% response rate in melanoma cases [131]. The failure of TIL treatments in some cancers has been attributed to the difficulty of isolating TILs from lesions, such as those in the lungs, liver, and brain, and most of the TILs in these cases were not sufficiently enriched to counteract the tumor-specific antigens [132].

Consequently, the second category of adoptive cell therapy (ACT) employed peripheral T cells isolated from patient blood, which were genetically engineered to express chimeric antigen receptors (CARs) capable of recognizing tumor-associated antigens present on major histocompatibility complex (MHC) molecules of cancer cells. This approach enabled the targeted killing of cancer cells and paved the way for the development of CAR T-cell therapy as a promising treatment for various cancers [133]. In a clinical trial, genetically engineered T cells expressing T-cell receptors (TCRs) specific for HLA A2 and targeting the MART1 peptide ligand were infused into refractory metastatic-melanoma patients. The infused T cells exhibited potent antitumor activity, resulting in the regression of the tumor in 30% of the patients. This finding underscores the therapeutic potential of TCR gene therapy in the treatment of cancer [134]. Additionally, CARs cells are generated by transducing patient T cells with virus-encoded specific DNA to establish the surface ligand of a monoclonal antibody or a co-stimulatory domain, such as CD28, to recognize specific surface antigens on tumor cells without the need for MHC molecules or APCs [135]. Retroviral-modified CAR T cells may also increase the release of cytokines, such as IFN -γ and TNF-α, as well as the release of perforin and granzyme granules in cytotoxic cells, to target tumor cells. The CAR-T-cell approach has achieved significant progress in immunotherapy because it overcomes tumors’ intrinsic mechanisms of resistance, such as the downregulation of MHC molecules, and induces a good response among resistant tumors, such as diffusing large-B-cell lymphoma [134].

Recent studies using animal models of colorectal cancer demonstrated the efficacy of CAR-T-cell therapies targeting guanylyl cyclase C protein (GUCY2C), a transmembrane protein expressed on the luminal surfaces of intestinal epithelial cells [22]. These findings represent a significant advancement in the development of CAR-T-cell therapies for colorectal cancer and also have implications for the treatment of gastric and esophageal cancers.

The third category of ACT involves harnessing the potential of NK cells, which are a type of innate immune cell isolated from peripheral blood. These NK cells are genetically modified and enriched to enhance their ability to target tumor cells, a process that is facilitated by the secretion of immune mediators that recruit and activate other immune cells, ultimately leading to a cytotoxic attack on cancer cells. This approach holds immense potential for cancer immunotherapy, and researchers are continuing to explore ways to optimize and fine-tune the use of NK cells in ACT [136]. This line was proven to be effective in trials in which 73% of CD19 cancer patients showed notable responses [137]. Adoptive cell therapy has demonstrated remarkable progress in clinical trials. However, a major current hurdle in this therapy is the need to mitigate the toxicities arising from immune cross-reactivity and the intense release of cytokines, including IL-6, IFN-γ, and TNF-α, a phenomenon referred to as cytokine storm [138].

Mesenchymal stem cells (MSCs) have emerged as promising tools in cancer therapy, owing to their unique biological properties and ease of isolation from various tissues, including bone marrow, adipose tissue, and umbilical-cord tissue [139]. These cells possess the ability to self-renew and differentiate into various cell types, including bone, cartilage, and adipose tissue. Furthermore, MSCs are known to secrete numerous growth factors and cytokines, which can increase tumor suppression, modulate the immune response, and enhance tissue repair. These properties make MSCs attractive candidates for cancer treatment, as they can be isolated and expanded in vitro with relative ease, without losing their functional characteristics, and can effectively target tumor tissues after systemic administration [139]. Mesenchymal stem cells have been found to have immunomodulatory properties, which means that they can influence the immune response in the TME by secreting cytokines and growth factors. This has been shown to support the development and proliferation of other immune and cancer cells, leading to a more effective antitumor response. In addition, genetically modified MSCs have been developed as promising tools for cancer therapy, particularly in drug and gene delivery. These modified MSCs have been engineered to express and secrete cancer-inhibitory agents, such as oncolytic viruses, chemokines, interferons, and transcription factors. This approach allows the targeted delivery of therapeutic agents to cancer cells while minimizing the toxicity and side effects associated with conventional chemotherapy. Ongoing research in this area aims to further optimize and refine the use of MSCs in cancer therapy and to develop more effective and targeted treatments for a range of malignancies [140].

### 8.5. Vaccines

The use of cancer vaccines is a promising approach to immunotherapy, with a wide range of products in development. These include cell-based vaccines, such as dendritic-cell vaccines, which present tumor-specific antigens to the immune system to activate an antitumor response. Virus-based vaccines, such as those using the adenovirus or poxvirus vectors, deliver tumor antigens to immune cells to stimulate an immune response against cancer cells. Nucleic-acid-based vaccines, such as DNA or RNA vaccines, encode tumor antigens to generate an immune response. Lastly, peptide-based vaccines contain short chains of amino acids that mimic tumor antigens and can be used to stimulate an immune response against cancer cells. Extensive research is underway to optimize the efficacy and safety of these various vaccine types, with the goal of developing effective cancer vaccines for a wide range of tumor types. Although cancer vaccines have shown promise in preclinical studies and early-phase clinical trials, significant challenges remain, including the need for better antigen selection, vaccine formulation, and strategies to overcome tumor-induced immune suppression [141]. The efficacy of these vaccines is built upon the crucial immunogenic antigens in cancers, including tumor-associated antigens (TAAs) and tumor-specific antigens (TSAs)/neo-antigens, to trigger the immune system [142,143]. Recently, clinical trials have shifted their focus towards exploring novel strategies that target both tumor-associated antigens (TAAs) and tumor-specific antigens (TSAs), in conjunction with immune checkpoint inhibitors (ICIs). This approach aims to improve immunotherapy response rates and prevent the development of resistance. In metastatic-melanoma patients, a significant reduction in tumors upon the administration of the neo-epitope RNA vaccine was found to prolong the survival of patients [144]. Further work is required to develop a novel cancer vaccine due to the complexity of the tumor microenvironment and the suppressive nature of chemokines and immune cells.

### 8.6. Exosomes

Exosomes are small extracellular vesicles that play a role in intercellular communication by transferring proteins, nucleic acids, and lipids among cells. Immune-cell-derived exosomes have gained attention in the study of cancer immunotherapy due to their ability to modulate the immune system. These exosomes can contain tumor-associated antigens, cytokines, and other immune-modulatory molecules, which can activate or suppress immune responses. Furthermore, exosomes can be modified to enhance their immunogenicity and antigen specificity by engineering them to express tumor-associated antigens or other immune-stimulatory molecules. For example, dendritic-cell-derived exosomes modified to express tumor antigens have shown promise in preclinical and early clinical studies as cancer vaccines. An early development was the use of APC-derived exosome prime-activated CD4^+^ and CD8^+^ T cells though the presentation of antigen-MHC class I/II molecules [130]. Subsequently, dendritic-cell-derived exosomes (DEX) and macrophages were used as cancer vaccines in various clinical trials, including trials on colorectal cancer and NSCLC [145].

Macrophages are immune cells that play a critical role in regulating the immune response in the tumor microenvironment. Exosomes released by macrophages have been shown to contain a variety of biologically active molecules, including inducible nitric-oxide synthase, reactive oxygen species (ROS), and pro-inflammatory cytokines, such as interleukin-6 (IL-6), interleukin-1 (IL-1), interleukin-23 (IL-23), and tumor necrosis factor alpha (TNF-α). These exosomes have been found to have antitumor effects through the induction of cancer-cell apoptosis. Therefore, the use of exosomes derived from macrophages holds great promise as a potential therapeutic strategy in cancer immunotherapy [146,147].

Dendritic-cell-derived exosomes also potentiate the function of both T-helper and cytotoxic T cells with ease of isolation and enrichment in vitro, with relative stability upon storage and with less toxicity compared to ACT cells. Thus, the use of cell-derived exosomes could be an alternative therapy in cancer treatment.

### 8.7. Epigenetic Modulators

In many human cancers, aberrant epigenetic modifications have been observed, which often contribute to cancer progression and immune evasion. Epigenetic changes generally regulate gene expression by altering DNA methylation, histone modifications, chromatin remodeling, glycosylation, ubiquitination, and noncoding RNAs. The epigenetic state of a cell determines its function, and DNA methylation is regulated by DNA methyl transferases (DNMTs). Recently, epigenetic modulators, such as DNMT inhibitors, have emerged as potential treatments for cancer by reversing DNA methylation. These DNMT inhibitors have been shown to be effective in cancer therapy as they reprogram specific tumor-suppressor genes or DNA-repair genes. These inhibitors can also enhance the immune response by upregulating the expression of immune-related genes and inhibiting the expression of immunosuppressive genes. Further research is required to fully understand the mechanisms underlying the epigenetic regulation of cancer and to develop more effective epigenetic therapies [148]. Examples of DNMT inhibitors include 5-Aza-CdR, which is an FDA-approved drug used to treat stage IV small-cell lung cancer; this drug partially improved the survival rates of patients due to toxicity and cross-reactivity [148].

Another epigenetic program that contributes to immune resistance and the progression of malignancies is the epithelial-cell–mesenchymal-cell transition (EMT), which encourages cancer development, invasion, metastasis, and resistance. Therefore, various clinical trials have illustrated the therapeutic potential of anti-EMT agents, such as anti-TGF-β, to impede tumors’ intrinsic resistance machinery and provoke the immune response [39]. Furthermore, lncRNAs (noncoding RNAs) inactivate the gene expression of epithelial cells and activate the mesenchyme-associated genes by inhibiting intracellular signaling pathways, such as JAK/STAT and TGF-ß/SMAD, and suppressing tumorigenesis [39].

### 8.8. Microbiota

The gut microbiota produces metabolites and small molecules to support human physiological processes, inflammation, immunity neurology, and metabolism. Recent research has demonstrated the gut microbiota as an immune-response modulator for T helper 1, CD8^+^ T cells, and tumor-associated myeloid cells [149]. This evidence has encouraged researchers to investigate the role of the gut microbiota in cancer treatment. Primarily, the microbiota was manipulated in combination with ICI therapy in preclinical and clinical cancer studies [150]. This included *Bacteroides Fragilies* and anti-CTLA-4 therapy in melanoma mice, resulting in better immune response [98]. Moreover, *Bifidobacterium* and PD-L1 blockade upregulates CD8^+^ cells, enhances DC maturation, and activates other immune cells in the TME [98]. In a clinical study of melanoma, fecal-microbiome transplantation (FMT) and pembrolizumab (anti-PD-1) provoked CD8^+^ T-cell activation in the TMEs of nonresponding melanoma patients through the modulation of several cytokines and chemokines that provoked resistance to anti-PD1, increasing the efficacy of and overcoming immune resistance to immunotherapy [151].

## 9. Overcoming Cancer Resistance to Immunotherapy

The development of novel immunotherapies has led to advancements in cancer treatment and has changed the survival rates of many cancer patients. While several agents have been approved by the FDA as monotherapies, including immune-checkpoint inhibitors (ICIs), which have revolutionized cancer treatment and promise durable cures, many patients suffer from what is known as “cold tumors”, with immune cells that infiltrate at low rates. Cancer cells have unique features that allow them to evade immune recognition by exploiting the TME, leading to resistance. To overcome this obstacle, a complete understanding of the complex molecular alterations in the TME must be obtained for each patient by analyzing the genetic makeup of cancer cells, as well as the abundant cytokines and immune cells present in the TME. Additionally, multiple approaches should be combined in multifaceted treatments to increase sensitivity to immunotherapy by harnessing different components within the immune system in favor of increasing the T-cell response. For example, in esophageal squamous cell carcinoma, one of the most common tumors in Eastern Asia, the use of dual therapy increased the overall survival in SCC patients up to 10.3 months compared to 6.7 months, which is the median survival rate with chemotherapy [152]. In recent years, researchers have also explored the potential of combining immunotherapy with other treatment modalities, such as chemotherapy, radiation therapy, and targeted therapy. For instance, preclinical studies have shown that combining ICIs with chemotherapy or radiation therapy can enhance antitumor immune responses by increasing the presentation of tumor antigens to immune cells and stimulating the release of pro-inflammatory cytokines. Additionally, targeted therapies that block specific signaling pathways involved in tumor growth and survival may also sensitize cancer cells to immune attack by decreasing the immunosuppressive factors in the TME. The use of atezolizumab–bevacizumab combination therapy in hepatocellular carcinoma showed a better overall survival rate than using separate therapies for HCC [153], in addition to the reversal of the immunosuppressive tumor microenvironment by combining ICIs with anlotinib, which alters the tumor immune microenvironment by downregulating PD-L1 expression on vascular endothelial cells.

Furthermore, advances in genetic-sequencing technology have allowed the identification of potential biomarkers that could be used to predict which patients will be most likely to respond to immunotherapy. For example, the expression of certain immune-related genes and the presence of specific immune-cell populations in the TME have been associated with better clinical outcomes in some cancer types. This information could help guide treatment decisions and improve patient selection for immunotherapy.

Overall, the development of novel immunotherapies and the combination of different treatment modalities hold great promise for improving outcomes in cancer patients, particularly those with previously untreatable or advanced disease. However, more research is still needed to better understand the complex interactions between cancer cells and the immune system and to identify new targets for immunotherapy that could overcome resistance mechanisms and lead to more durable responses.

In Table 1, several examples of approved combinations of immunotherapies are listed, highlighting the improvements in response.

## 10. Future Directions for Cancer Immunotherapy

Immunotherapy has revolutionized cancer treatment by harnessing the power of the immune system to fight cancer cells. It has shown remarkable success in treating various types of cancer, including melanoma, lung cancer, and bladder cancer. However, many challenges still need to be addressed to improve the effectiveness of immunotherapy and expand its use to more cancer types. The following is a discussion of some of the future directions of immunotherapy.

## 11. Combination Therapies

The combination of different types of immunotherapy agent and combining immunotherapy with other treatments, such as chemotherapy or radiotherapy, have shown promising results in preclinical and clinical studies. For example, combining anti-PD-1/PD-L1 agents with anti-CTLA-4 agents has shown improved response rates in patients with melanoma and renal-cell carcinoma [164]. Similarly, combining immunotherapy with targeted therapy agents, such as BRAF inhibitors, has shown improved outcomes in patients with BRAF-mutant melanoma [165]. Furthermore, combining immunotherapy with local therapies, such as radiation therapy, can enhance the immune response by releasing tumor antigens and stimulating immune-cell infiltration [166]. Therefore, combination therapies are likely to be a major subject of research on immunotherapy in the future.

## 12. Personalized Immunotherapy

The success of immunotherapy depends on the patient’s immune system and the unique characteristics of their tumor. Therefore, the development of personalized immunotherapy strategies that consider the patient’s immune status and tumor characteristics is likely to be a major direction of research on immunotherapy in the future. For example, the tumor-mutation burden (TMB) and microsatellite instability (MSI) have been shown to be predictive biomarkers for the response to immunotherapy [167]. Therefore, developing personalized immunotherapy strategies based on these biomarkers could improve patient selection and treatment outcomes.

## 13. Adoptive Cell Therapy

Adoptive cell therapy (ACT) involves harvesting T cells from the patient, expanding and activating them in vitro, and infusing them back into the patient to target and kill cancer cells. Adoptive cell therapy has shown remarkable success in treating hematological malignancies, such as leukemia and lymphoma [168]. However, its effectiveness in solid tumors is limited by the hostile tumor microenvironment and the lack of specific tumor antigens. Therefore, developing strategies to enhance the efficacy of ACT in solid tumors is likely to be a major direction of research on immunotherapy in the future. One approach is to genetically modify T cells to express chimeric antigen receptors (CARs) that can target specific tumor antigens [169]. Another approach is to use T-cell receptor (TCR) gene therapy to redirect T cells to recognize tumor antigens.

## 14. Overcoming Resistance

Despite the remarkable success of immunotherapy, not all patients respond to treatment, and some develop resistance over time. Therefore, the development of strategies to overcome resistance to immunotherapy is likely to be a major direction of immunotherapy research in the future. Resistance to immunotherapy can occur through various mechanisms, including the loss of tumor-antigen expression, the upregulation of immune-checkpoint molecules, and the activation of alternative signaling pathways. Therefore, developing combination therapies that can target multiple resistance mechanisms is likely to be an effective strategy to overcome resistance to immunotherapy.

## 15. Novel Immunotherapy Agents

Finally, developing novel immunotherapy agents that can target new pathways and mechanisms is likely to be a major direction of immunotherapy research in the future. For example, bispecific antibodies that can simultaneously target two different antigens on cancer cells and T cells have shown promising results in preclinical and early clinical studies [170]. Similarly, immune agonists that can activate specific immune pathways, such as the STING (stimulator of interferon genes) pathway and the NLRP3 (NOD-like receptor family pyrin domain-containing 3) inflammasome pathway, have shown potential in enhancing the antitumor immune response [171].

In conclusion, immunotherapy has revolutionized cancer treatment, and in the future, research on immunotherapy is likely to focus on improving its effectiveness and expanding its use to more cancer types. Combination therapies, personalized immunotherapy, adoptive cell therapy, overcoming resistance, and the development of novel immunotherapy agents are some of the major directions of immunotherapy to be explored in the future. With ongoing research and development in these areas, we can expect to see further improvements in the outcomes of cancer treatment and in the quality of life of cancer patients.

## Figures and Tables

**Table 1:** Clinically approved combined immunotherapies in various cancers.

Treatment	Modalities of Combined Immunotherapy	Tumor Type	Phase	Clinical Trial	Ref.
Durvalumab + autologous anti-CD19CAR-4-1BB-CD3z EGFRt-expressing CD4+/CD8+ central memory T lymphocytes JCAR014	Anti- PDL-1 + CAR T cells	Diffuse large B-cell lymphoma	1	NCT02706405	[154]
Pembrolizumab + CART-EGFRvIII T cells	Anti- PD-1 + CAR T cells	Glioblastoma	1	NCT03726515	[11]
Inupadenant + EOS-448	Adenosine A receptor blocker + anti-TIGIT mAB	Advanced solid tumors	2	NCT05060432	[155]
Ciforadenant ± atezolizumab	Adenosine A receptor blocker + anti-PD-1	Advanced prostate cancer	1,1b	NCT02655822	[156]
Ciforadenant + daratumumab	Adenosine A receptor blocker + mAb against CD38	Multiple myeloma	1	NCT04280328	[157]
AB928/etrumadenant + zimberelimab + enzalutamide	Adenosine A and B receptor blocker + anti-PD-1 + anti-androgen or chemotherapy	Advanced prostate cancer	2	NCT04381832	[158]
AB928/etrumadenant zimberelimab + AB680	Adenosine A and B receptor blocker + anti-PD-1 + CD73 inhibitor	Colonic cancer	2	NCT04660812	[158]
Etrumadenant +PLD + IPI-549	Adenosine A and B receptor blocker + chemotherapy + PI3K-gamma inhibitor	Breast cancer	1	NCT03719326	[159]
Bintrafusp alfa	PD-L1 and TGF β inhibitors	Squamous cell carcinoma	1	NCT02699515.	[11,152]
CD19 CAR T-expressing IL7 and CCL19 combined with tislelizumab	IL17, CCL9 chemokine, and PD-1 mAb	Relapsed or refractory B-cell lymphoma	1	NCT04381741	[160]
HuMax-IL8 + nivolumab	CXCL8-chemokine and PD-1- IC	Head and neck squamous	2	NCT04848116	[123]
Entinostat plus pembrolizumab	Histone deacetylase inhibitors plus PD-1 blockade	Non-small-cell lung cancer (NSCLC)	2	NCT02437136	[161]
Autogene cevumeran and atezolizumab	mRNA-based cancer vaccine and anti-PD-L1	Metastatic tumors	1	NCT03289962	[162]
Azacitidine and PD-L1 inhibitors	Demethylating agent plus PD-L1 inhibitor	Acute myeloid leukemia	2	NCT02775903	[163]
